# Apprehending anxiety: an introduction to the Topical Collection on worry and wellbeing

**DOI:** 10.1007/s11229-022-03794-9

**Published:** 2022-08-01

**Authors:** Juliette Vazard, Charlie Kurth

**Affiliations:** 1 City University of New York, Graduate Center and University of Geneva, Geneva, Switzerland; 2 Department of Philosophy, Helsinki Collegium for Advanced Studies and Western Michigan University, Michigan, USA

## Abstract

The aim of this collection is to show how work in the analytic philosophical tradition can shed light on the nature, value, and experience of anxiety. Contrary to widespread assumptions, anxiety is not best understood as a mental disorder, or an intrinsically debilitating state, but rather as an often valuable affective state which heightens our sensitivity to potential threats and challenges. As the contributions in this volume demonstrate, learning about anxiety can be relevant for debates, not only in the philosophy of emotion, but also in epistemology, value theory, and the philosophy of psychopathology. In this introductory article, we also show that there is still much to discover about the relevance that anxiety may have for moral action, self-understanding, and mental health.

Consider anxiety: In spite of the ubiquity of anxiety in our lives, few within analytic philosophy have discussed its intentional structure, the value it may have for our moral and epistemic lives, or its phenomenology.^1^ The aim of this collection is to show how work in the analytic tradition can shed light on the nature, value, and experience of anxiety and related negative emotions. As the contributions of this collection reveal, understanding the nature of anxiety is not only of interest to debates in the philosophy of emotion – on, say, the relation between emotions and moods or on the conditions of fittingness of emotions – it’s also of interest to debates in epistemology and value theory regarding the role of emotions for intellectual and moral virtues, not to mention debates in the philosophy of psychology and psychopathology.

Negative emotions – and perhaps anxiety particularly – have traditionally been presented as inherently detrimental, even impairing, affective states. Unfortunately, most of the research on anxiety has painted a picture of it as a debilitating or pathological mental state. This might mislead us into thinking that “anxiety” only refers to a mental disorder, or that it is an intrinsically impairing affective state. While the literature both in philosophy and psychology has tended to focus on cases in which anxiety manifests to an intense degree and with deleterious effects, moderate anxiety generally proves beneficial, both by enhancing our performances, and by sensitizing us to all sorts of potential dangers and threats. Indeed, as philosophers increasingly explore the value and contribution of a wider and wider range of emotional states, the idea that states like anxiety are entirely useless appears less and less plausible (Kurth, 2018a; Miceli & Castelfranchi, 2015; Barlow, 2001). And thus, after a long history of neglect in both folk conceptions and philosophy, anxiety is now starting to be acknowledged as an important feature of our psychology. In this volume, we therefore largely refer to anxiety, not as a clinical phenomenon, but rather as the occurrent affective state which is part and parcel of the everyday lives of healthy individuals.

As we will see, emerging philosophical work on anxiety’s moral, aretaic, and epistemic value suggests that when anxiety is felt in a way that is fitting, rational, or appropriate to one’s situation, it can be an important element of the good life. But the prevalence of anxiety disorders and the deeply unpleasant, sometimes paralyzing feelings that accompany anxiety indicate anxiety also has a darker side. Recognizing this raises important – but underexplored – questions regarding the range of our anxiety experiences and the role that conceptual and empirical work might play in distinguishing adaptive from maladaptive forms of anxiety.

In what follows, we begin with a brief sketch of how anxiety is typically understood in emotion research, noting some of the central normative and evaluative questions that philosophical work on anxiety and related emotions raises (§ 1). We then discuss four themes that structure the “Worry and Wellbeing” collection, briefly summarizing the contribution of each essay with respect to these themes (§ 2). We then conclude with some indications of where we see new philosophical work on anxiety heading (§ 3). As will become apparent, the essays in this collection understand labels like “anxiety,” “worry,” and “wellbeing” in a very broad way. That was intentional: we wanted the collection to engage philosophers thinking about these topics from a variety of different perspectives. We think the result is a rich set of papers offering a compelling introduction into the philosophy of anxiety.

## What is anxiety and why does it Matter?

An idea that guides much of our thinking about emotion maintains that any viable understanding of the value of a given emotion must be grounded in an understanding of the underlying nature of that emotion. But, as we’ll see, developing an adequate understanding of emotions like anxiety is difficult in large part because of the diversity in the ways in which the term “anxiety” gets used. Thus, we begin with a brief overview of some of this complexity before turning to talk more specifically about how anxiety and its value have typically been understood in the philosophical and empirical literatures.

### Making sense of our anxiety talk

In thinking about the nature of anxiety, the first central philosophical question we can raise is: what kind of phenomenon is “anxiety”? .First of all, in virtue of its phenomenological and motivational features, anxiety seems best classified as an *affective* phenomenon, and distinguished from non-affective states (such as, for instance, judgements and assumptions). But what kind of affective phenomenon is anxiety – an emotion, a mood, a feeling? Or all of these? In practice, psychologists, psychiatrists and philosophers tend to use the term “anxiety” to cover a variety of phenomena that would be difficult to group around a common core. Despite the absence of a clear category, this volume testifies to the fact that there is much to be said about the affective state of anxiety, beyond the disorders and excessive but non-clinical ways in which it manifests itself.

The broad category of affective phenomena can be said to include, among other things, emotions, moods, feelings, and dispositions or character traits. Our affective life is composed of a variety of phenomena, which may manifest as occurrent episodes that are experienced (such as emotions and moods), or as dispositions to experience these occurrent affective episodes. Where should we locate anxiety within this typology? If we consider anxiety as an emotion – i.e. a short-lived episode directed at a specific intentional object – we need to specify its intentional structure. What are the typical objects that anxiety is directed at? How does anxiety evaluate these objects (i.e. as carrying an evaluative property of some sort)? How do we apprehend a situation when we respond to it with anxiety?

Recent accounts of anxiety, both in psychology and philosophy, have helped refine our view of the intentional structure of anxiety (Kurth, 2018a; Miceli & Castelfranchi, 2015). In the psychological literature, anxiety has been defined as an emotion through which we evaluate an event or situation as implying a possible and uncertain threat (Miceli & Castelfranchi, 2005, 2015). In a similar vein, Charlie Kurth argues that anxiety is an emotion which tracks and responds to cues of “problematic uncertainty” (2015, p. 5). This formulation captures what seem to be the two essential evaluative components of anxiety: the lack of information on the part of the subject (an uncertainty), and the fact that this uncertainty is “problematic,” given that it concerns a possible threat, challenge, or thwarting of one’s goals. Beyond questions regarding the specific intentional objects of anxiety as an emotion, we can ask how this phenomenon relates to the phenomenon of anxious *mood*. As we will see in § 2.4, the volume addresses the distinctions and interactions between anxiety as an emotion and anxiety as a mood. How is the intentional structure of an anxious mood different from the intentional structure of an episode of anxiety? How do both of these phenomena relate to the emotion of fear, and to pathologies such as anxiety disorders?^2^

### Puzzles about anxiety’s value

A second important question concerns the value of anxiety, though it has been largely understudied both in the psychological and in the philosophical literature. What good, if any, might anxious experiences bring? How does anxiety contribute to our wellbeing, both personal and interpersonal? If anxiety is (or can be) valuable, in what ways does this value manifest? Is anxiety only instrumentally valuable, or might experiencing anxiety also have a final value?

There’s little doubt on the fact that anxiety carries the instrumental value of preparing us to better cope with potential threats. The way in which we apprehend possibilities of danger largely relies on affect (Slovic & Peters, 2006), and certain emotions function to render danger and risk salient, allowing us to prepare for the emergence of possible threats. This is the case of a variety of emotions such as fear, anxiety, apprehension, worry, dread, terror, panic, etc. (Roberts, 2003). By rendering risk and danger salient, these emotions give priority to the goal of avoiding harm, and orient our decision-making accordingly. Anxiety warns us of potentially dangerous situations and helps us react more quickly and robustly to protect what we care about (Barlow, 2001; Kurth, 2018a: Chap. 4). Just as anger or guilt take on specific shapes in order to help us deal with the types of problems they signal, anxiety involves vigilance to threats and automatic action tendencies of risk minimization and risk assessment. Thus, while it is clear that our ability to experience anxiety in the face of uncertain threats and challenges has instrumental value, does it also have final value? Might it be intrinsically good for us to feel anxiety?

Let us be clear, we are not denying that anxiety can have detrimental effects, such as generating epistemically biased reasoning (like jumping to catastrophic conclusions) and prudentially harmful behavior. Rather, we are suggesting that, while those detrimental effects have been amply studied (e.g., Tuma & Maser 2019), the value and benefits of anxiety have received far less attention. This being said, over the last decade or so, researchers have helped refine our understanding of anxiety’s value, particularly its value in the epistemic and moral domains. How might experiencing anxiety help us achieve epistemic success or maintain our moral standing? A handful of authors have suggested that anxiety can be intrinsically valuable by contributing to epistemic success (Hookway, 1998; Nagel, 2010; Vazard,^2021^), virtuous agency (Kurth, 2018a, b), moral decision making (Lacewing, 2005; Kurth, 2015), and our sense of ourselves (Ratcliffe, 2008). Anxiety, it has been argued, is an emotion that allows us to reliably achieve both practical, moral, and epistemic goods. An appropriate disposition to feel anxiety in the moral and in the epistemic domains, and thus to attend to and anticipate both moral and epistemic mistakes, might even be said to constitute virtuous character traits. Virtues are stable and successful dispositions that reliably help us achieve some good, which can be epistemic, moral, or prudential (Turri et al., 2019). In this sense, virtues are finally valuable. However, virtues can also be considered to have further value insofar as they are an important source of well-being for an individual (Haybron, 2008). If anxiety is a form of awareness or attunement to possible threats of various kinds, it might be an emotional faculty that grounds both our tendencies towards moral concern and epistemic caution (Roberts & Wood, 2007). Insofar as it is through feeling anxiety that we apprehend situations as implying problematic uncertainties, the character trait of cautiousness can be understood as grounded in a disposition to experience appropriate and well-calibrated anxiety. In this sense, anxiety could be viewed as a virtuous disposition which reliably contributes to our epistemic and moral successes. If this is valid, our disposition to feel anxious in the right situations and to the right degree might even constitute an important source of well-being for us. We will explore these hypotheses in the various themes we now introduce.

## Four foundational themes of the Collection

With an initial understanding of anxiety and its (potential) value in hand, we turn to discuss four central themes in philosophical work on anxiety and related emotions, which structure the eleven contributions to the Worry and Wellbeing collection.

### Anxiety’s epistemic value

Recently, an affective revolution has taken place in epistemology, as philosophers now recognize the importance of emotions for reasoning, inquiry, and belief formation. Indeed, it has recently been argued that emotions can be a valuable aid to our everyday questioning and investigation, as well as in scientific inquiry (Hookway, 2002, 2003; Thagard, 2002, Brun et al., 2008, de Sousa 2009). Thagard (2002), for example, provides a comprehensive typology of the role of emotions in scientific research. More generally, it is now widely acknowledged that many important cognitive functions could not be performed successfully without relying on the emotions (e.g. de Sousa 1987; Lazarus, 1991; Frijda et al., 2000). Certain emotions in particular are thought to play a distinct role in our attempts to acquire beliefs that we have reasons to form, or reassess beliefs we have reasons to doubt. These emotions or feelings, sometimes referred to as “epistemic emotions,” constitute a controversial and diverse category. Whether there really are specifically epistemic emotions, and what emotions this category includes, are questions still being debated today.

Within these debates, the emotions which have most often taken center stage are typically positive emotions – emotions such as curiosity, surprise, wonder, and interest. Curiosity is a state that has attracted the interest of both philosophers of emotion and epistemologists (Whitcomb, 2010; Williamson, 2002, p. 31; Inan 2013; Kvanvig, 2012), who have investigated its psychological nature and its role in our motivation to inquire and ultimately gain valuable information. More recently, psychologists and philosophers have started to consider the role of negative emotions – such as confusion or frustration – in our ability to acquire knowledge (Silva, 2010; Vazard & Audrin 2021). Nonetheless, it is clear that anxiety, by triggering a state of vigilance and caution, gears up our attention, reasoning, and inference-making so as to help us anticipate threats and their consequences. Anxiety is thus associated with a general orientation of our attention, motivation, and thought patterns; and, of course, the specific way in which anxiety drives all of these facets of cognition in turn impacts our epistemic aims and activities.

Several of the essays in this volume are specifically focused on investigating how an emotional disposition to feel anxiety might contribute to a subject’s ability to acquire knowledge. If anxiety helps us stay vigilant and alert to potential epistemic threats, how might we conceive of anxiety’s epistemic value? Is there such a thing as “epistemic anxiety”? If so, should we view it as (or part of) an epistemic virtue? And what does this state inform us of, exactly? In the intellectual domain, anxiety (understood either as a character trait or as a disposition), can be equated with what Robert Roberts and W. Jay Wood (2007) call “intellectual caution” (p. 219) and, as with moral virtue, anxiety so understood is an important trait of the epistemically virtuous, one that contributes to intellectual virtues such as vigilance, care, and conscientiousness (Montmarquet, 1992; Zagzebski, 2005).

Like all human activities, intellectual practices (such as research, investigation, inquiry, etc.) involve significant threats: one might be careless in one’s reasoning and belief-forming processes, or in focusing one’s attention on non-relevant sets of information. If so, then there seems to be many ways in which a disposition to experience anxiety could be of great help to the virtuous intellectual. For instance, a state of vigilance and caution, applied to the context of inquiry, might ensure that the agent both remains sensitive to epistemic threats (such as false beliefs) and motivated to guard against them (Roberts & Wood, 2007). Additionally, being able to distinguish real from perceived epistemic threats, and to manage the resulting fear and anxiety, is constitutive of the epistemically virtuous individual.

Recent literature in both the philosophy of emotion and epistemology indeed suggests that anxiety is an epistemically valuable emotion: it positively contributes to the success of many of our epistemic endeavors. On this front, Hookway (1998) turned philosophers’ attention to the role of the affective signal of anxiety in our ability to immediately detect “unsafe” beliefs – beliefs which we should not rely on but instead reassess. To this, Nagel (2010) adds that “epistemic anxiety” is a psychological phenomenon which allows us to better calibrate the cognitive resources we invest in forming beliefs based on the stakes in play. Juliette Vazard (2021) has hypothesized that if epistemic anxiety is responsible for our adaptive inclinations to doubt and gather further evidence on high-stakes matters, then an excess of epistemic anxiety might be involved in certain forms of dysfunctional doubting or epistemic over-cautiousness – what we see, for instance, in patients with obsessive–compulsive disorder.

The phenomenon of “epistemic anxiety” has also attracted the attention of several philosophers contributing to this volume. In “Epistemic Anxiety and Epistemic Risk”, Lilith Jane Newton formulates an account of “epistemic anxiety” in terms of epistemic risk: the risk of believing in error. In an attempt to illuminate the relationship between anxiety and risk, Newton asks: how should risk be conceived if it is to be the kind of thing that can elicit anxiety as an emotional response? Proposing a refinement to existing accounts (specifically, Nagel 2010 and Vazard, 2021), Newton considers three accounts of risk: normic (in which risk is determined by the most normal worlds in which a negative event obtains), probabilistic risk (determined by the likelihood of its obtaining), and modal (determined by the closeness of worlds in which a negative event obtains). She argues that epistemic anxiety is a response to epistemic risk triggered by normic and probabilistic, but not modal risk.

In her own contribution, Juliette Vazard looks at the phenomenon of “Everyday Anxious Doubt” and examines the role of anxiety in our everyday tendencies to question our beliefs. Although the relation between anxiety and doubt has already been highlighted (Hookway, 1998, 2008), there has been little effort to elaborate on the psychological mechanisms through which an affective state like anxiety generates a motivation to reassess our beliefs. Elaborating on Neil Levy’s account of the epistemic effects of “Everyday Anxiety” (2016), Vazard provides a model of the kind of emotion-cognition interactions at play in our everyday anxious doubts. Additionally, she shows how clarifying the role of anxiety in these phenomena helps us revise a common assumption about the interactions between anxiety and higher-level cognitive processes, such as the ones involved in representing hypothetical threatening scenarios through mental imagery.

Moving towards virtue epistemology, Frank Cabrera proposes that developing a virtuous disposition to feel the appropriate amount of epistemic anxiety works to promote epistemic success and so contributes to intellectually virtuous inquiry. In “Is Epistemic Anxiety an Intellectual Virtue?”, Cabrera asks whether epistemic anxiety counts as an epistemic virtue in the sense familiar to reliabilist epistemologists. In developing an affirmative answer to this question, Cabrera argues that central aspects of inference to the best explanation reasoning – in particular, our ability to recognize that some fact requires an explanation and to be motivated to employ this form of inference – are both facilitated by feelings of epistemic anxiety. As Cabrera sees it, recognizing that a fact requires an explanation is a species of practical knowledge; as such, securing such knowledge requires the agent to develop the virtuous disposition to feel the appropriate amount of epistemic anxiety.

As the contribution of anxiety to our epistemic endeavors is slowly being uncovered, another area of study concerns the value of experiencing an affective state like anxiety in the context of our moral and prudential goals.

### Anxiety’s practical value

The thought that anxiety might have practical value is often met with skepticism – and there’s good reason for this. For one, anxiety seems to be an emotion that undermines thought and action. So, for instance, we see Kant counseled against strong negative emotions on the grounds that they make reflection “impossible or more difficult” (1797/1996: 535). Ask skepticism of this sort can also be found in the Stoics’ assessment of emotions (especially negative ones ) as problematic (Kurth, 2018a, c). Work in psychology brings some empirical support to these worries. For instance, a recent review of research investigating the effects of anxiety in evaluative settings notes that it’s “predominantly harmful to task performance” (Zeidner & Matthews, 2005: 147). At a more familiar level, we have the tales of anxiety-driven disasters in popular audience books and biographies. Consider, for instance, what we find in Scott Stossel’s memoir *My Age of Anxiety* (2013). Stossel gets anxious whenever he needs to speak before a large group. While such public speaking anxiety is a fitting response given the risk of negative social judgment that such settings bear, Stossel’s anxiety brings such intense cycles of dread, nausea and sweating that he must resort to Xanax and vodka to prevent himself from running out on the talk he’s supposed to give. Hardly a picture of anxiety contributing to health and wellbeing.

But this familiar picture is not the whole of anxiety’s story. For every Stossel-like anxiety disaster, we can point to a case where anxiety brings a valuable attunement and responsiveness to the various threats pending on our cherished goods. Consider, for instance, the suffragist Elizabeth Cady Stanton. In her autobiography (1898/1993), Stanton recounts the anxiety that she felt about getting married. While her father vehemently opposed her marriage because her fiancé was an abolitionist, Stanton found herself captivated by her husband-to-be’s principles and the impassioned anti-slavery speeches she’d seen him give. Still, the anxiety Stanton felt about this conflict was intense and, at one point, it even led her to call off her engagement. But those worries also prompted her to seek advice from her sister about what she ought to do. Not only did those anxiety-driven reflections help Stanton see that she should renew her plan to get married, but they also revealed why she *should* do it and what she valued. For instance, shortly after she renewed her marriage plans, Stanton protested her minister’s suggestion to include a traditional vow of obedience in the ceremony. As she explained, a vow to obey was fundamentally at odds with the equal union she was entering into with her soon to be husband.

Similarly, Nelson Mandela often remarked on the unease that the demands of being both a father and a freedom fighter brought. In fact, these anxieties led him to reflect on “whether one was ever justified in neglecting the welfare of one’s own family to fight for the welfare of others” (1994, p. 212). As with Stanton, Mandela’s anxiety reveals his sensitivity to holding important, but sometimes clashing, values. Were he not anxious about how to reconcile his competing obligations to his family and the anti-apartheid movement, our admiration of him as a moral exemplar would diminish. Moreover, Mandela’s anxiety also helped him clarify his thinking. He came to see that his commitments – to both family and the fight for freedom – were equally important and deeply intertwined. Fighting for freedom was, in some way, fighting for his family (Kurth, 2020; Lacewing, 2005).

But, of course, you don’t need to be a suffragist or freedom fighter from the history books to benefit from anxiety. A moderate twinge of helpful anxiety is a common feature of everyday life. The pinch of unease felt when talking to a new acquaintance signals that you may have said something offensive; this discomfort then brings an increased deference that can help you get your conversation back on track.

But the result of these observations is a mess. We don’t have a clear understanding of whether (or when) an emotion like anxiety has practical value. In thinking about this issue, philosophers have tended to focus on a pair of questions: When is anxiety a fitting or rational response? And how does the value of one’s fitting anxiety trade-off against one’s overall wellbeing and prudential interests?^3^ Two papers in the collection take up these issues.

First, in his article, “Fitting Anxiety and Prudent Anxiety,” James Fritz highlights a tension between the conditions in which it is *fitting* to experience anxiety, and the conditions in which it is *prudent* to experience anxiety. While the norms for fitting anxiety are concerned with whether a situation merits feeling the emotion, the norms of prudence are concerned with whether feeling an instance of anxiety will promote our wellbeing. As Fritz argues, anxiety provides perhaps the clearest case study on the tension between norms of fittingness and prudence. An enormous array of possible outcomes are both threatening and uncertain. While it might be fitting to feel anxiety in all these situations, it is plausibly deeply imprudent to demand that we actually do that. Thus, when it comes to anxiety, Fritz concludes that fittingness norms seem to stand in an inescapable conflict with the norms of prudence.

In contrast to Fritz, Heidi Maibom’s paper, “Don’t Worry, Be Happy?” defends a more optimistic answer to questions about our ability to be fittingly anxious without undermining other things we care about – like our own wellbeing. As she shows, the issue here is delicate. On the one hand, a failure to effectively regulate anxiety leads to things like less happiness, less success, and worse health. But, on the other hand, anxiety is increasingly recognized as a morally relevant response to others’ suffering. But this suggests that using emotion regulation to maximize aspects of happiness might *reduce* one’s moral goodness. In an effort to alleviate this tension, Maibom argues that the apparent conflict between cultivating anxiety and cultivating happiness can be resolved. But seeing how this can be done requires us to consider anxiety in its context, and in relation to the other affective states which arise alongside it, as well as the significance of the overall experience to the agent.

### Regulating and calibrating anxiety

Implicit in the above discussions of anxiety’s epistemic and practical value is the idea that whether an instance of anxiety is valuable or not turns on whether it is felt at the right time and in the right way. This in turn raises questions about what, if anything, we can do to regulate – or cultivate – emotions like anxiety.

Here it might be useful to say a little about how we can distinguish emotion cultivation from emotion regulation. Briefly, emotion cultivation is a term that is more common in philosophy (especially in the context of work on virtue in the Aristotelian tradition). It refers to our intentional efforts to bring lasting changes to when and how we experience a particular emotion so as to promote certain values (prudential, moral, epistemic, etc.).^4^ By contrast, talk of emotion regulation is more prevalent in psychology. It typically refers to our unconscious efforts to affect short-term changes in when and how we experience an emotion in a given situation for hedonic reasons (e.g., to perpetuate the good feeling of positive emotions and curtail the bad experience of negative ones).^5^

Looking more closely at emotion cultivation, we find philosophical discussions of it going back to the ancients, and the central issues taken up then continue to shape current debates. This includes questions like: Can we shape our emotions for the better? If so, should that be a goal of our educational practices and efforts to promote moral development? What cognitive capacities are central to emotion cultivation and what can we do to develop them? Is emotion cultivation best understood as an individualistic process or a more social/institutional one?

In the context of anxiety, fear, and similar phenomena, clinical psychology and emotion science licenses modest optimism about our ability to cultivate these emotions. For instance, cognitive-behavioral therapy and the use of “fear appeals” show promise as methods that can help correct false-positives (feeling anxious when one shouldn’t) and false-negatives (not feeling anxious when one should) (Hofmann & Smits, 2008; Abramowitz, 2001; Keller, 1999; Lewis et al., 2007). In a similar vein, reappraisal techniques have proven effective moderating the appropriate, but excessive anxiety experiences when, say, taking a test (Jamieson, Mendes, et al., 2010; Hanin, 2007; Hatzigeorgiadis & Biddle, 2001). That said, there is still much work we to do in order to better understand the theoretical and normative questions that underlie these empirical findings.

On this front, several essays in the collection can be seen as contributing to our understanding of how we might effectively regulate and cultivate complex emotions like anxiety. For instance, Ditte Marie Munch-Jurisic argues in, “Lost for Words: Anxiety, Well-being, and the Costs of Conceptual Deprivation,” that while negative affective states such as distress, discomfort, and anxiety can indeed be morally and epistemically valuable, whether an individual will be able to realize these benefits depends on her capacity to correctly and constructively interpret her own affective states. Particularly, Munch-Jurisic points out that an agent’s socio-economic and cultural context may prevent her from developing the hermeneutic equipment (words, concepts, etc.) necessary to make sense of the negative affective states she is experiencing in these contexts, thereby depriving her of a tool that might be essential to improving her wellbeing and mental health.

In a similar vein, Heidi Maibom’s “Don’t Worry, Be Happy?” asks how we can cultivate morally valuable forms of anxiety and do so in ways that do not undermine our happiness. Her answer is that we’re likely to do better in cultivating emotions like anxiety to the extent that we focus less on the particular instances of the emotion and more on how our anxious experiences evolve over time and interact with other affective experiences (empathy, hope, etc.) and our perceptions of our situations.

In contrast to the focus on an individual’s anxious experiences that we see in the papers from Munch-Jurisic and Maibom, several other contributors argue that attaining a richer understanding of anxiety requires us to move beyond an individualistic perspective on emotions. For instance, in “The Relational Calibration of Fear,” Ami Harbin considers fear from an interpersonal perspective. How does fear shape an agent’s relationships in times of crises and how, in turn, is an agent’s fear shaped by those relationships? Integrating results from empirical psychology, Harbin provides an account of the role of interpersonal relationships in emotional processes of fear. How do other agents – both those we trust and those we don’t – impact our responses to danger in the context of a collective crisis such as the COVID-19 pandemic?

Focusing on a specific kind of interpersonal relationships, Troy Jollimore’s, “Anxious Feelings, Anxious Friends: On Anxiety and Friendship,” examines the phenomenon of proxy anxiety – that is, the anxiety that friends feel on our behalf. Moving beyond first-personal cases of anxiety, or how one’s emotions impact one’s own wellbeing, Jollimore examines several ways in which our friends’ reactions of anxiety might benefit our own affective functioning and wellbeing. How might the emotions that my friends experience contribute positively to my own wellbeing? In short, our friends’ distinctive relationships with us leave them well-positioned to help us regulate our ill-calibrated anxieties.

Finally, Charlie Kurth’s contribution, “Inappropriate Emotions, Marginalization, and Feeling Better,” examines an objection – namely, that calls to cultivate or correct “inappropriate” emotions like anxiety, anger, and shame can lead to further marginalization of the already marginalized. Not only do these emotions tend to be experienced more often by women, minorities, and other marginalized members of our communities, but the emotion norms that dictate who can feel what and when too often work to entrench power relations that benefit dominant group members. Drawing on research in cognitive science examining how we acquire and deploy the norms that shape our behavior, the paper sketches a proposal for how we might cultivate emotions in ways that can help us avoid marginalizing the already marginalized.

### The experience of anxious states and what this explains

A final important question concerns the broader nature of anxious states and what it is like to experience them. The affective life of an individual is made up of a variety of phenomena, which may manifest themselves in the form of experienced episodes, or of dispositions to experience such episodes. Anxious states thus comprise not only the emotion of anxiety (i.e., a short-lived episode directed at a specific state of affairs), but also anxious moods. In fact, in the philosophical literature, it is the mood state of anxiety that philosophers have historically been most interested in (Kierkegaard, 1844/2006; Heidegger et al., 1962). Moods, like emotions, are associated with bodily sensations and feelings (they have a specific phenomenology), but they typically last for longer. Moreover, although this is a controversial idea, it is often argued that, while emotions have intentional objects, moods do not seem to target specific objects (Lormand, 1985; Kurth, 2022: Chap. 3). Thus, while emotions often call for reasons and can be considered correct or incorrect, we do not tend to think of moods as justified (or correct) or unjustified (or incorrect). How does anxiety manifest when it takes the form of a mood? And how does anxiety as a mood relate to anxiety as an emotion? Finally, does anxious mood have a specific value or contribution to our wellbeing in any way?

One author of this volume addresses these questions and provides an original account of the relation between anxious mood and anxious emotion, as well as a view of the psychological benefits of such an interaction between the different anxious states. In “Affective Shifts: Mood, Emotion and Well-being,” Jonathan Mitchell provides a detailed philosophical account of affective shifts – when our moods “crystalize” into emotions, and our emotions “diffuse” into moods – applied to anxiety. Anxiety can manifest in the form of a short-lived emotional episode that is intentionally directed at a specific event or situation, but also in the form of an anxious mood whose intentionality is less focused and more diffuse. Mitchell proposes that these two states are often connected, and that the way in which moods ‘develop’ into emotions can, in certain cases at least, allow for a kind of ‘release from affect’, which may be important for maintaining proper affective functioning.

Finally, we turn to a further dimension of the felt experience of anxiety and its impact on wellbeing – namely, its relevance to understanding symptoms of psychopathology. Just 40 years ago, anxiety did not exist as a clinical category in its own right. In 1927, according to the Psychological Abstracts list, only three scientific papers on anxiety were published, and by 1950 there were only 37. It wasn’t until 1980 – after new medications designed to treat anxiety were developed and brought to market – that anxiety disorders were finally introduced into the third edition of the *American Psychiatric Association’s Diagnostic and Statistical Manual of Mental Disorders* (DSM-III), replacing the Freudian neuroses to which anxiety had previously belonged. It is thus now recognized that anxiety can take different pathological forms, and manifest in ways that significantly impair a subject’s functioning. Anxiety and its associated disorders is indeed now thought to represent the most common form of officially listed mental illness in the United States (Simpson et al., 2010; Kessler et al., 2005) and the European Union (Wittchen et al., 2011).

Clinical, chronic anxiety is distinct from the occasional episodes of anxiety that are part of the life of healthy individuals. Not only do these anxious episodes manifest with an incredibly excessive intensity, but they also target inappropriate objects by arising in situations – on ordinary days, doing ordinary things – which present no apparent menace. While we have seen that anxiety often promotes individual functioning, pathological anxiety (as well as non-pathological but severe anxiety) can lock a subject into rigid and paralyzing cycles of rumination and worries, and leave them in a constant state of hypervigilance to threats. One author of this volume addresses the nature and phenomenology of rumination for patients with depression and pathological anxiety. In “Stuck on Repeat: Why do we Continue to Ruminate?” Jodie Louise Russell examines the phenomenon of rumination and asks: How does rumination develop for patients with depression or pathological anxiety? And how can we account for the phenomenological features of these repetitive kinds of thought? Building on Lisa Feldman Barrettt’s theory of emotion categorization as sense-making, and on Merleau-Ponty’s work, Russell develops an integrated model of rumination that takes into account both underlying bodily processes and the lived experience of the ruminator.

## Future directions in philosophical work on worry and wellbeing

Studies pointing to significant increases in public anxiety levels suggest that we may be turning to a new “age of anxiety.” Regardless of whether that is the case, it’s clear that anxiety accompanies and sustains many human activities and experiences, and that it deserves further study. In this concluding section, we highlight four areas we believe to be ripe for philosophical exploration.

The first line of study concerns anxiety’s relationship to other similar emotions. Many articles have touched on the relationship between anxiety and other affective states of “bad prospects,” such as fear and worry. However, anxiety shares interesting features with positive emotions too. Particularly, anxiety belongs to the small class of “emotions of uncertainty” which require that one does not know the relevant facts about the object of one’s emotion. Hope is another interesting emotion of uncertainty (Gordon, 1969, 1987), which is positively valenced, but likewise involves that one does not take oneself to know whether, and under which circumstances, the event that one is hoping for will get realized. Recognizing this reveals that the relationship between hope and anxiety is tighter than one might think: it is very common for one to sway between those two states in situations of uncertainty, in which we hope for the best but often get anxious that the worst ends up happening (Vazard, forthcoming). Anxiety and hope thus appear to be our two primary ways of being emotionally engaged with the unknown. This raises several interesting philosophical questions. What is the value of maintaining a balance between hope and anxiety in times of uncertainty? How do the two emotions work together to help us stay both motivated and alert, vigilant but determined, so that our overall affective engagement towards the future is one which allows us to skillfully navigate uncertainty?

On this front, research on “eco-anxiety” and other ecological emotions – that is, emotions experienced in the context of climate change and other ecological crises – may offer insight. Despite some headlines suggesting that eco-anxiety is a pervasive and too often pernicious emotion (Hickman et al. 2021; BBC 2019), work by environmental researchers suggests that eco-anxiety is often a fitting response: the eco-anxious are worried about something that really is worrisome (Ojala et al., 2021; Kurth & Pihkala, forthcoming). Related work adds that eco-anxiety can foster pro-environmental behaviors (e.g., buying green products, becoming involved in environmental activism). Moreover, this research also suggests that these resulting pro-environmental behaviors are most pronounced when individuals experience (moderate) amounts of anxiety *combined* with hope (Ojala, 2012; Nabi et al., 2018; Kurth and Pihkala, forthcoming). But research on eco-anxiety is just in its infancy and much of the empirical work is under-theorized, offering an opportunity for philosophers to contribute to our understanding of thorny questions. What, for instance, is eco-anxiety? In what ways, if any, might it be a fitting or valuable response to climate change? How should we understand, both conceptually and causally, the relationship between eco-anxiety and eco-hope? Are eco-emotions responses that we can cultivate and, if so, how might we go about that?

On the matter of cultivation, while many have noted that the value of negative emotions like anxiety, anger, fear, and shame is intimately bound up with our ability to shape them for the better, less has been done to explain what this amounts to or why it might be plausible to think that anxiety (and the like) is a psychological state we might be able to shape. Granted, there is work in clinical psychology and psychiatry that serves, in a sense, as “proof of concept” – the improvements that treatment protocols bring licenses optimism that these emotions can be substantively changed for the better (e.g., Kurth 2018a, 2018c; Liebow & Glazer, 2019). There is also work in virtue ethics that discusses our general ability to cultivate our emotions (e.g., Sherman 1989; Annas, 2011; cf., Kurth 2021). But as the essays here make plain, there is much work still to be done. In particular, as of yet, the relevance of empirical work on anxiety (and emotion) regulation is at best suggestive of our ability for cultivation—that is, our ability to affect long-term change in when and how we experience a given emotion. Moreover, since many of the core concepts here (e.g., emotion regulation, emotion cultivation) and developmental goals (e.g., should we aim to transform, attune, or just control problematic emotions?) are under theorized, here too there is much that philosophers can contribute.

Third, there is the distinct, but related, project of investigating connections between emotions like anxiety and self-knowledge and self-understanding. Within the continental tradition, anxiety has often been seen as a – if not the – emotion that is central to our understanding of who we are (e.g., Kierkregaard 1844/2006; Sartre 1943; Ratcliffe, 2008; Tietjen, 2020). What new light might analytical philosophy bring to this question? Insofar as anxiety alerts us of situations which we view as problematic (and therefore important), could anxiety constitute an important way to get in touch with our actual personal values, priorities, and goals? By noticing those areas of our lives which, when they find themselves threatened, make us feel anxious, we have an avenue for gaining an understanding of what we view as worth protecting. In other words, the idea would be that if one wants to find out what one truly cares about, one should perhaps first and foremost look at what makes one anxious.

Finally, anxiety and fear underlie many emotional disorders, and there is a considerable degree of comorbidity between anxiety disorders such as generalized anxiety disorder and social anxiety disorder, obsessive-compulsive disorder, or phobias. Sufferers are likely to experience more than one category across a lifetime (Brown & Barlow, 1992). For this reason, it is plausible that one of the benefits of further understanding the nature of anxiety is that it could help us better understand what is similar between, and what is distinct in, these disorders. The nosographic classifications of disorders are imperfect and evolving, and this is an area where philosophical methods of conceptual analysis can help.

## Footnotes

1 By contrast, philosophers working in the continental tradition have extensively examined the nature, value, and phenomenology of anxiety. See, for instance, Kierkegaard 1844/2006; Sartre 1943; Heidegger et al., 1962; Ratcliff 2008; Shockey 2016.

2 To date, little philosophical work has examined the relationship between anxiety (the emotion) and anxious moods (but see Kurth 2018a: 9–11; Kriegel 2019; Price, 2006). On anxiety’s connection to fear, see (e.g.) Kurth 2018a: Chap. 2 and Öhman 2008.

3 A variety of authors have wrestled with questions of how fitting forms of anxiety might trade-off against other values. See, for instance, Hookway 1998; Kurth, 2018a, 2018b, 2016; Tietjen, 2020; Summers & Sinnott-Armstrong, 2015.

4 For recent discussion of emotion cultivation within the context of (Aristotelian) virtue ethics see Sherman 1989, Hursthouse, 2001, Kristjánsson, 2018, and Kurth 2018a, 2021.

5 Within psychology, James Gross’s work is foundational (e.g., Gross 2015). Also see, Aldao 2013 and Tamir 2016.
